# Evolution of patterns of care for women with cervical cancer in Morocco over a decade

**DOI:** 10.1186/s12885-022-09358-x

**Published:** 2022-05-02

**Authors:** Abdellatif Benider, Karima Bendahhou, Catherine Sauvaget, Hind Mrabti, Farida Selmouni, Richard Muwonge, Leila Alaoui, Eric Lucas, Youssef Chami, Loubna Abousselham, Maria Bennani, Hassan Errihani, Rengaswamy Sankaranarayanan, Rachid Bekkali, Partha Basu

**Affiliations:** 1 Centre Mohammed VI Pour Le Traitement Des Cancers, Centre Hospitalier Universitaire Ibn Rochd de Casablanca, Casablanca, Maroc; 2 Registre Des Cancers de La Région du Grand Casablanca, Casablanca, Maroc; 3 Early Detection, Prevention & Infections Branch, International Agency for Research On Cancer (WHO), 150 cours Albert Thomas, 69372 Cedex 08 Lyon, France; 4 Institut National d’oncologie, CHU-Rabat, Rabat, Morocco; 5 National Cancer Institute, Fes, Morocco; 6 Lalla Salma Foundation, Prevention and Treatment of Cancers, Rabat, Morocco; 7 Cancer Unit, Epidemiology and Disease Control Department, Ministry of Health, Rabat, Morocco; 8 RTI International India, New Delhi, India

**Keywords:** Cervical cancer, Pattern of care, Treatment delay, Morocco, Disease-free survival

## Abstract

**Background:**

We conducted a Pattern-of-care (POC) study at two premier-most public-funded oncology centers in Morocco to evaluate delays in care continuum and adherence to internationally accepted treatment guidelines of cervical cancer.

**Method:**

Following a systematic sampling method, cervical cancer patients registered at Centre Mohammed VI (Casablanca) and Institut National d’Oncologie (Rabat) during 2 months of every year from 2008 to 2017, were included in this retrospective study. Relevant information was abstracted from the medical records.

**Results:**

A total of 886 patients was included in the analysis; 59.5% were at stage I/II. No appreciable change in stage distribution was observed over time. Median access and treatment delays were 5.0 months and 2.3 months, respectively without any significant temporal change. Concurrent chemotherapy was administered to 57.7% of the patients receiving radiotherapy. Surgery was performed on 81.2 and 34.8% of stage I and II patients, respectively. A very high proportion (85.7%) of operated patients received post-operative radiation therapy. Median interval between surgery and initiation of radiotherapy was 3.1 months. Only 45.3% of the patients treated with external beam radiation received brachytherapy. Radiotherapy was completed within 10 weeks in 77.4% patients. An overall 5-year disease-free survival (DFS) was observed in 57.5% of the patients – ranging from 66.1% for stage I to 31.1% for stage IV. Addition of brachytherapy to radiation significantly improved survival at all stages. The study has the usual limitations of retrospective record-based studies, which is data incompleteness.

**Conclusion:**

Delays in care continuum need to be further reduced. Increased use of chemoradiation and brachytherapy will improve survival further.

**Supplementary Information:**

The online version contains supplementary material available at 10.1186/s12885-022-09358-x.

## Background

Stage-appropriate evidence-based treatment of at least 90% of patients with cervical cancer is one of the major pillars supporting the World Health Organization (WHO) strategy for cervical cancer elimination. Pattern-of -care (POC) studies are conducted to assess the status of dissemination of evidence-based practices at healthcare settings routinely delivering oncology care and also to evaluate the impact of such practices [1, 2]. Evidence-based management of cervical cancer underwent a major change during the turn of the century with role of concurrent chemoradiation and that of high dose rate (HDR) brachytherapy being strongly established. There is sparse data from low- and middle-income countries (LMICs) on adoption of these best practices in cervical cancer management, even though 80% of global burden of the disease is borne by these countries [3].

Morocco as an LMIC from Middle East North Africa (MENA) region made significant investments in the last decade in improving cancer care services [4]. National breast and cervical cancer screening programmes were launched in the year 2010 [5]. Treatment facilities for cervical cancer were significantly improved at the premier public-funded oncology centers in the country—Centre Mohammed VI pour le traitement des cancers (CM-VI) in Casablanca and Institut National d’Oncologie Sidi Mohamed Ben Abdellah (INO) in Rabat. Iridium^192^ high dose rate (HDR) brachytherapy was installed in 2009 at both centers. By 2010, all the telecobalt machines were replaced by dual energy linear accelerators (LINAC) and all patients treated with LINAC were expected to undergo individual dosimetry.

We report in the present manuscript the outcomes of a POC study focusing on cervical cancer management in Morocco jointly implemented by International Agency for Research on Cancer (IARC) France, Ministry of Health, Morocco and Lalla Salma Foundation for Cancer Prevention and Treatment, Morocco. The aim of the study was to evaluate the socio-demographic characteristics of the patients, stage distribution and pathology types, delays in care pathway, quality of treatment by stages and its impact on disease-free survival. Through retrospective inclusion of patients registered between the years 2008 and 2017 we aimed to study the temporal trends in different variables.

## Method

The retrospective study was conducted at CM-VI, Casablanca and INO, Rabat. At the initiation of our study these were the only comprehensive cancer management public facilities in the country with oncosurgery, chemotherapy, 3D conformal radiation therapy (RT) and HDR brachytherapy. However, there were differences in organization of services at the two centers. While INO had all services under a single roof with a single management structure, some of the critical services (pathology and laboratory services, radiology, oncosurgery) at CM-VI were delivered at the adjacent tertiary care University Hospital. Though both CM-VI and University Hospital were public-funded Institutions, their governance were different.

Patients with histopathologic diagnosis of cervical cancer registered at the two oncology centres between 2008 and 2017 were eligible for inclusion. Diagnostic confirmation could have happened before or after registration at the centre. Patients with recurrent cervical cancer at the time of registration were excluded. We used a systematic sampling method rather than including all patients registered during the study period. Eligible patients registered during a 2-month period of each year, starting from 2008 and ending in 2017, were recruited. The bimonthly sampling cycle started in January and February for 2008, shifted to the next 2 months each year, and restarted in January and February after 6 years. The last sampling was in June and July 2017.

Case files of the patients with cervical cancer were obtained from the medical records department at the two hospitals and scanned for information by trained staff (a PhD student at CM-VI and a research nurse at INO). Data was abstracted in a data collection form designed and pretested to collect demographic information, pathology reports, staging, treatment details (surgery, radiotherapy, chemotherapy) and follow-up status. The project staff were supervised at each hospital by the institutional principal investigator. The completed data collection forms were entered in an online dedicated database. The entered data could be regularly checked by a coordinator at IARC for completeness, consistency, and validity.

Distribution of the patient characteristics was presented as proportions, stratified by the period of diagnosis (2008–2012 and 2013–2017). The clinical stage classification by International Federation of Gynaecologists and Obstetricians (FIGO) was used. The centers introduced the FIGO 2009 classification in the same year [6]. The effect of different patient characteristics on advanced stage (stage-III/IV) at registration was assessed and presented as odds ratios (ORs), obtained from posterior distribution median and their confidence intervals from the 2.5 and 97.5 percentiles of the Bayesian logistic regression model.

Disease recurrence after treatment was the only outcome that we could assess in the survival analysis. Overall survival couldn’t be estimated as majority of deaths happened outside the oncology centres and information was not documented in case records. Endpoint in the disease-free survival (DFS) analysis was defined as being found alive with disease (relapse) during follow-up. Only patients who underwent cancer-directed treatment (surgery, radiation or chemotherapy) were considered. Follow-up time for DFS was measured starting from the date of treatment initiation for all patients. The end date was the date of relapse for patients who experienced the endpoint. For patients without any documented relapse the endpoint was date of death or date of last follow up, whichever was earlier. Kaplan–Meier estimates were presented for probability of relapse over the study duration.

The frequencies for the patient characteristics assessed and Kaplan Meier curves were done in Stata 15.1 (StataCorp LP, Texas, USA). The Bayesian regression models were carried out using Just Another Gibbs Sampler (JAGS) software [7, 8]. JAGS was used in order to additionally model for the missing data in the outcomes and/or explanatory variables [9]. The study was approved by the ethics committees at IARC and the participating institutions. A waiver of informed consent was obtained for the retrospective study.

## Results

Data was abstracted from case-records of total 907 patients registered at the two institutions following the sampling plan. On subsequent scrutiny, a few recurrent cases and cases without histopathology confirmation were identified and excluded. A total of 886 patients (CM-IV: 352; INO: 534) were included in the final analysis. Majority of the patients (60.3%) were registered at INO. A decline in the number of registered cervical cancer patients with time was observed at both the centers.

Patient characteristics, stage at registration and histopathological types of cancer by the year of registration (stratified as 2008–12 and 2013–17) are shown in Table 1. The median age at diagnosis was 55 years (IQR: 48–64 years); age distribution remained similar across the two time periods. More than one third (34.0%) of the patients were premenopausal. Stage information was available for 787 (88.8%) patients; 59.5% of them were detected at FIGO stage I or II. No temporal variation was observed in stage distribution. The median interval between symptom onset and first consultation with a health professional leading to the diagnosis (‘access delay’) was 5.0 months (IQR: 3.0–10.0), with modest (not statistically significant) improvement over time [2008–12: 6.0 month (IQR: 3.0–10.0); 2013–17: 5.0 months (IQR 3.0–11.5)]. On multivariate analysis, only access delay and period of registration were significantly associated with advanced stage of cancer (Supplementary Table 1). In contrast, parity appeared to have a protective effect on advanced cancer stage (Supplementary Table 1). As the duration of access delay increased by 1 month, the likelihood of being diagnosed at an advanced stage significantly increased by 1.8% (95% CI: 0.4–3.2%). Proportion of stage III/IV patients was higher among those registered in 2013–17 compared to previous years.

**Table 1:** Cervical cancer patient characteristics by period of registration

Characteristics	Period of registration		Total
	**2008–2012**	**2013–2017**	**n (%)**
	**n (%)**	**n (%)**	
Patients assessed	504	382	886
Centre			
CM-VI, Casablanca	208 (41.3)	144 (37.7)	352 (39.7)
INO, Rabat	296 (58.7)	238 (62.3)	534 (60.3)
Age at registration (years)			
< 50	150 (29.8)	125 (32.7)	275 (31.0)
50–59	169 (33.5)	133 (34.8)	302 (34.1)
60 +	185 (36.7)	124 (32.5)	309 (34.9)
Total	504 (100.0)	382 (100.0)	886 (100.0)
Residence			
Urban	337 (66.9)	264 (69.1)	601 (67.8)
Semi-urban	45 (8.9)	48 (12.6)	93 (10.5)
Rural	122 (24.2)	70 (18.3)	192 (21.7)
Total	504 (100.0)	382 (100.0)	886 (100.0)
Parity			
0–2	111 (23.1)	87 (25.4)	198 (24.1)
3–4	134 (27.9)	92 (26.8)	226 (27.5)
5 +	235 (49.0)	164 (47.8)	399 (48.5)
Total	480 (100.0)	343 (100.0)	823 (100.0)
Missing	24 (11.5)	39 (27.1)	63 (17.9)
Menopause status			
Premenopausal	175 (36.2)	99 (30.7)	274 (34.0)
Postmenopausal	308 (63.8)	224 (69.3)	532 (66.0)
Total	483 (100.0)	323 (100.0)	806 (100.0)
Missing	21 (10.1)	59 (41.0)	80 (22.7)
Diagnosis confirmed before registration at oncology centre	468 (92.9)	345 (90.3)	813 (91.8)
FIGO stage			
I	43 (9.0)	23 (7.4)	66 (8.4)
II	255 (53.6)	147 (47.3)	402 (51.1)
III	159 (33.4)	108 (34.7)	267 (33.9)
IV	19 (4.0)	33 (10.6)	52 (6.6)
Total	476 (100.0)	311 (100.0)	787 (100.0)
Missing	28 (5.6)	71 (18.6)	99 (11.2)
Tumour type			
SCC	463 (92.0)	333 (87.9)	796 (90.2)
Adenocarcinoma	24 (4.8)	23 (6.1)	47 (5.3)
Others	16 (3.2)	23 (6.1)	39 (4.4)
Total	503 (100.0)	379 (100.0)	882 (100.0)
Missing	1 (0.2)	3 (0.8)	4 (0.5)
Tumour differentiation			
Well differentiated	182 (40.2)	70 (24.4)	252 (34.1)
Moderately differentiated	213 (47.0)	152 (53.0)	365 (49.3)
Poorly differentiated	54 (11.9)	57 (19.9)	111 (15.0)
Others	4 (0.9)	8 (2.8)	12 (1.6)
Total	453 (100.0)	287 (100.0)	740 (100.0)
Missing	51 (10.1)	95 (24.9)	146 (16.5)

*CM-VI* Centre Mohammed VI pour le traitement des cancers (CM-VI), *INO* Institut National d’Oncologie Sidi Mohamed Ben Abdellah, *FIGO* International Federation of Gynecologists and Obstetricians, *SCC* Squamous Cell Carcinoma

The median interval between diagnosis of cancer and initiation of treatment (treatment delay) was 2.3 months (IQR 1.5–3.4), without any significant improvement observed over the years [2008–12: 2.3 months (IQR: 1.4–3.3 months); 2013–17: 2.4 months (IQR: 1.6–3.8 months)] (data not shown in tables). A significant reason for treatment delay was long waiting period at the hospitals to initiate treatment. The median delay for treatment initiation after registration was 1.6 months (IQR: 0.9–2.3 months) in 2008–12 and 1.7 months (IQR 1.2–2.7 months) in 2013–17.

Treatment information was available for 789 (89.1%) patients; 68 of them didn’t have stage information. Details of treatment by stage are described in Table 2.

**Table 2:** Treatment received by stage at diagnosis by cervical cancer patients registered during 2008–2017

Treatment received	Patients	Stage at diagnosis				
	assessed	I	II	III	IV	Unknown
	n (%)	n (%)	n (%)	n (%)	n (%)	n (%)
Patients registered	886	66	402	267	52	99
Patients without treatment details	97 (10.9)	2 (3.0)	21 (5.2)	25 (9.4)	18 (34.6)	31 (31.3)
Patients with treatment details	789 (89.1)	64 (97.0)	381 (94.8)	242 (90.6)	34 (65.4)	68 (68.7)
Treatment type						
Surgery alone	38 (4.8)	15 (23.4)	11 (2.9)	2 (0.8)	2 (5.9)	8 (11.8)
Radiotherapy alone	199 (25.2)	8 (12.5)	93 (24.4)	75 (31.0)	7 (20.6)	16 (23.5)
Radiotherapy and chemotherapy only	325 (41.2)	4 (6.3)	150 (39.4)	135 (55.8)	13 (38.2)	23 (33.8)
Surgery and radiotherapy only	105 (13.3)	28 (43.8)	64 (16.8)	5 (2.1)	1 (2.9)	7 (10.3)
Surgery, radiotherapy and chemotherapy	90 (11.4)	9 (14.1)	55 (14.4)	15 (6.2)	1 (2.9)	10 (14.7)
Surgery and chemotherapy only	3 (0.4)	0 (0.0)	1 (0.3)	1 (0.4)	0 (0.0)	1 (1.5)
Chemotherapy alone	29 (3.7)	0 (0.0)	7 (1.8)	9 (3.7)	10 (29.4)	3 (4.4)
Radiotherapy type						
EBRT and brachy therapy	326 (46.1)	20 (41.7)	189 (53.1)	90 (39.5)	4 (18.2)	23 (43.4)
EBRT alone	322 (45.5)	16 (33.3)	136 (38.2)	131 (57.5)	17 (77.3)	22 (41.5)
Brachy therapy alone	59 (8.3)	12(25.0)	31 (8.7)	7 (3.1)	1 (4.5)	8 (15.1)

*EBRT* External Beam Radiotherapy

Radical hysterectomy was performed among 81.2% (52/64) of patients with stage I and 34.4% (131/381) of patients with stage II disease. Many of the surgeries were performed outside the two oncology centers. The proportion was less in 2013–17 (29.5%) compared to 2008–12 (49.3%).(data not shown in tables) Vast majority of the total operated patients (195/236; 82.6%) received post-operative RT. The median interval between surgery and initiation of RT was 3.1 months (IQR: 2.2–4.9 months), without any appreciable change over time [2008–12: 3.2 months; (IQR: 2.1–4.9); 2013–17: 3.0 months (IQR: 2.2–4.8)].

RT with concomitant chemotherapy (chemoradiation) was used to treat 6.3% (4/64) patients belonging to stage I, 39.4% (150/381) patients belonging to stage II, 55.8% (135/242) patients belonging to stage III and 38.2% (13/34) patients belonging to stage IV. Overall, 719 patients received RT, and of them only 415 (57.7%) received concomitant chemotherapy.

Further details of RT by the time periods are described in Table 3. The proportion of patients receiving RT was similar between 2008–12 (92.6%; 437/472) and 2013–17 (89.0%; 282/317). Brachytherapy was administered after external beam RT only among 45.3% (326/719) patients receiving RT; the proportion being lower in 2013–17 (35.5%; 100/282) compared to 2008–12 (51.7%; 226/437). Overall, 8.2% (2008–12: 8.0%; 2013–17: 8.5%) of the patients treated with RT received brachytherapy alone. The proportion of patients receiving HDR brachytherapy was significantly higher in 2013–17 (89.5%; 111/124) compared to 2008–12 (31.8%; 83/261) (Table 3).

**Table 3:** Radiotherapy details of cervical cancer patients treated during 2008–2017

	**Period of diagnosis**		**Total**
	**2008–2012**	**2013–2017**	**n (%)**
	**n (%)**	**n (%)**	
Patients who received treatment	472	317	789
Patients who received radiotherapy	437 (92.6)	282 (89.0)	719 (91.1)
EBRT and brachytherapy	226 (51.7)	100 (35.5)	326 (45.3)
EBRT alone	171 (39.1)	151 (53.5)	322 (44.8)
Brachytherapy alone	35 (8.0)	24 (8.5)	59 (8.2)
Unknown	5 (1.1)	7 (2.5)	12 (1.7)
Patients who received brachytherapy	261	124	385
Brachytherapy type			
Low dose rate	178 (68.2)	13 (10.5)	191 (49.6)
High dose rate	83 (31.8)	111 (89.5)	194 (50.4)
Patients who received Concomitant CT-RT (chemoradiation)	233 (53.3)	147 (52.1)	380 (52.9)
If radiotherapy was received before or after registration at oncology centre			
Before	20 (4.6)	23 (8.5)	43 (6.1)
After	413 (95.4)	247 (91.5)	660 (93.9)
Missing	4 (0.9)	12 (4.3)	16 (2.2)
EBRT total	397	251	648
Duration (weeks)			
< 4	58 (15.7)	21 (9.5)	79 (13.4)
4–6	198 (53.5)	113 (51.1)	311 (52.6)
7–8	44 (11.9)	22 (10.0)	66 (11.2)
9 +	70 (18.9)	65 (29.4)	135 (22.8)
Total duration of radiotherapy (weeks)			
< 10	137 (85.1)	48 (61.5)	185 (77.4)
10–12	9 (5.6)	10 (12.8)	19 (7.9)
13–14	7 (4.3)	7 (9.0)	14 (5.9)
15 +	8 (5.0)	13 (16.7)	21 (8.8)
Hospitalization for radiotherapy	61	9	70

*EBRT* External beam radiation therapy, *CT-RT* Concurrent chemo–Radiation therapy

Percentage of treated cervical cancer patients receiving chemoradiation was similar in 2008–12 (53.3%; 233/472) and 2013–17 (52.1%; 147/317). Cisplatin was the most frequently used chemotherapeutic agent used in 87.7% (355/405) of the patients receiving chemoradiation. (data not shown in tables).

Total time required for completion of EBRT alone and time required to complete full course of RT are described in Table 3. Overall, 66% of the patients completed EBRT within 6 weeks and 77.4% of the patients completed full course of radiation in less than 10 weeks. The proportion of patients for whom the total duration of RT exceeded 10 weeks was higher in 2013–17 (25.7%) compared to that in 2008–12 (14.9%). Only 9.7% (70/719) patients required hospitalization during RT and the proportion was significantly less in the later period (3.2%).

**Fig. 1:**
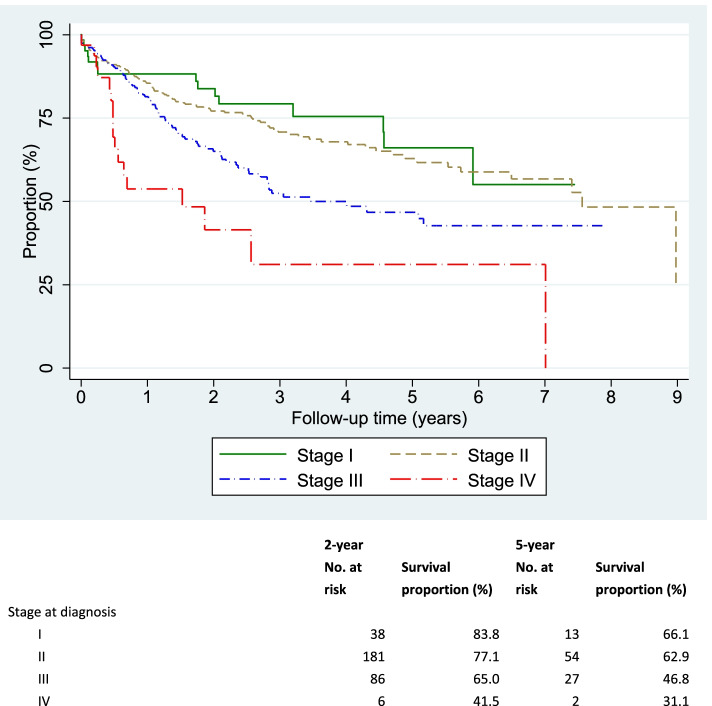
Kaplan Meier’s curve showing disease-free survival to relapse after treatment among cervical cancer patients treated during 2008–2017 by stage at diagnosis

**Fig. 2:**
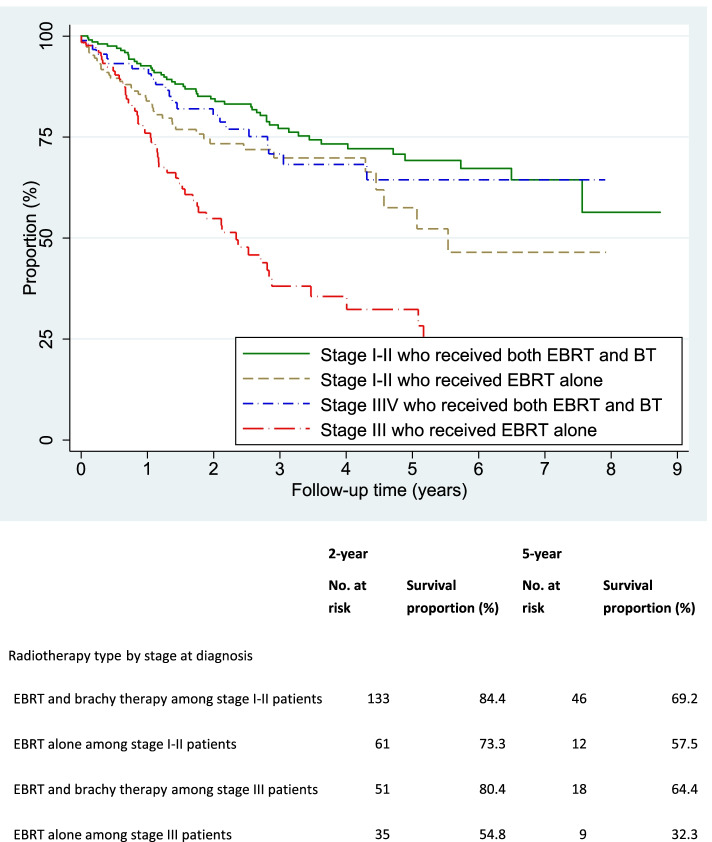
Kaplan Meier’s curve showing disease-free survival to relapse after treatment among cervical cancer patients treated during 2008–2017 by stage at diagnosis and if patients received both external beam radiotherapy (EBRT) and brachy therapy or EBRT alone

Overall, 95.1% (750/789) of the treated patients had at least one documented follow up visit at the oncology centers. Of them, 58.1% (N = 461) were free of any disease at last follow up. The median interval between treatment initiation and last follow up visit was 1.5 years (IQR 0.5–3.0 years). The 5-year DFS was 57.5% among the treated patients – ranging from 66.1% for stage I to 31.1% for stage IV (Fig. 1). Increasing age, advancing stage, being treated outside the oncology centers were significant independent factors influencing 5-year DFS on multivariate analysis (Table 4). The patients treated at CM-VI had significantly lower DFS compared to those treated at INO even after adjusting for age and other factors. Patients receiving brachytherapy along with EBRT had significantly higher survival compared to those treated with EBRT alone, irrespective of stages of cancer (Fig. 2).

**Table 4:** Independent determinants of disease-free survival among cervical cancer patients treated during 2008–2017

	**Patients who received treatment ** **n**	**Person- years of observation (PYO) ** **n**	**Patients who had disease relapse ** **n**	**Hazard rate per 100 PYO ** **(95% CI)**	**Crude hazard rate ratio ** (95% CI)*	**Adjusted hazard rate ratio ** (95% CI)**
Overall	773	1,669	227	13.6 (11.9 - 15.5)		
Centre						
CM-IV, Casablanca	261	547	125	22.8 (19.2 - 27.2)	1.00	1.00
INO, Rabat	512	1,122	102	9.1 (7.5 - 11.0)	0.65 (0.52 - 0.79)	0.35 (0.26 - 0.46)
Period						
2008–2012	467	1,195	168	14.1 (12.1 - 16.4)	1.00	1.00
2013–2017	306	474	59	12.4 (9.6 - 16.1)	0.80 (0.59 - 1.03)	0.78 (0.54 - 1.05)
p for trend						0.065
Age at diagnosis (years)						
< 50	253	552	63	11.4 (8.9 - 14.6)	1.00	1.00
50–59	257	637	77	12.1 (9.7 - 15.1)	0.87 (0.68 - 1.09)	1.07 (0.69 - 1.53)
60 +	263	480	87	18.1 (14.7 - 22.3)	1.26 (0.98 - 1.53)	1.44 (0.86 - 2.24)
p for trend						0.032
Place of residence						
Urban	524	1,142	146	12.8 (10.9 - 15.0)	1.00	1.00
Semi-urban	84	161	22	13.7 (9.0 - 20.7)	1.08 (0.64 - 1.58)	1.38 (0.76 - 2.10)
Rural	165	366	59	16.1 (12.5 - 20.8)	1.10 (0.82 - 1.40)	0.97 (0.68 - 1.31)
Parity						
0–2	172	338	54	16.0 (12.2 - 20.9)	1.00	1.00
3–4	192	466	55	11.8 (9.1 - 15.4)	0.87 (0.64 - 1.13)	0.81 (0.52 - 1.21)
5 +	359	820	106	12.9 (10.7 - 15.6)	0.96 (0.78 - 1.16)	0.78 (0.51 - 1.10)
p for trend						0.370
Menopausal status						
Pre	253	604	74	12.3 (9.8 - 15.4)	1.00	1.00
Post	462	977	134	13.7 (11.6 - 16.3)	1.01 (0.83 - 1.19)	0.93 (0.61 - 1.35)
Stage at diagnosis						
I	63	167	15	9.0 (5.4 - 14.9)	1.00	1.00
II	376	913	101	11.1 (9.1 - 13.4)	0.80 (0.65 - 0.97)	1.35 (0.74 - 2.27)
III	236	451	83	18.4 (14.8 - 22.8)	1.28 (1.00 - 1.56)	2.64 (1.44 - 4.54)
IV	33	37	17	46.1 (28.7 - 74.2)	2.87 (1.20 - 4.57)	6.07 (2.05 - 12.12)
p for trend						< 0.001
Tumour type						
Squamous cell carcinoma	699	1,524	208	13.6 (11.9 - 15.6)	1.00	1.00
Adenocarcinoma	39	68	13	19.2 (11.2 - 33.1)	1.25 (0.59 - 2.01)	1.42 (0.67 - 2.36)
Others	31	75	5	6.7 (2.8 - 16.1)	0.50 (0.14 - 1.03)	0.51 (0.13 - 1.08)
Tumour differentiation						
Well differentiated	222	559	72	12.9 (10.2 - 16.2)	1.00	1.00
Moderately differentiated	331	690	101	14.6 (12.0 - 17.8)	1.03 (0.83 - 1.25)	1.32 (0.93 - 1.80)
Poorly differentiated	97	228	29	12.7 (8.8 - 18.3)	0.91 (0.59 - 1.24)	1.09 (0.64 - 1.64)
p for trend						0.563
When treatment carried out						
All after registration	642	1,372	194	14.1 (12.3 - 16.3)	1.00	1.00
Both before and after registration	104	265	24	9.1 (6.1 - 13.5)	1.70 (0.09 - 4.79)	2.72 (0.02 - 11.54)
All before registration	26	30	8	26.3 (13.2 - 52.6)	7.51 (4.03 - 11.75)	13.45 (2.82 - 35.23)
Duration of symptoms (months)					1.00 (0.99 - 1.01)	1.00 (0.99 - 1.01)

*CM-IV* Centre Mohammed VI pour le traitement des cancers, *INO* Institut National d'Oncologie Sidi Mohamed Ben Abdellah, *CI* Confidence Interval

* adjusted for clustering within the centre; ** all listed variables included in the adjusted regression model

## Discussion

The POC study in Morocco reveals some major gaps in comprehensive care of the cervical cancer patients. Though a few advancements in quality of care was documented in more recent years (modest reduction in access delay, higher proportion of patients being operated at the comprehensive cancer centers, higher proportion of patients being treated with HDR brachytherapy etc.), there are scopes for improvement in several areas. Total number of cervical cancer patients registered at either center was less in the second 5-year period (2013–17) compared to the previous one. This is most likely attributed to the new regional oncology centers built in other regions during the second phase sharing the caseload. A reduction in estimated number of new cervical cancers in the country over time has been reported by IARC GLOBOCAN database [3]. Reduction in the number of new cervical cancer patients at the two oncology centers may be partly ascribed to the temporal trend in incidence of the cancer.

Median age at diagnosis of cervical cancer in our study was commensurate with the global average of 53 years and that reported from other LMICs [10]. In higher resourced countries the age-specific incidence of cervical cancer does not rise significantly after the age of 40 years due to the preventive impact of screening and the age-specific incidence peaks around 40 years of age [11]. Unfortunately, this phenomenon is not observed in LMICs having very limited cervical cancer screening opportunities. The same is applicable to Morocco. Though a VIA-based opportunistic cervical cancer screening programme was launched in the country in 2010, formal evaluation of the program in 2015–16 reported several deficiencies, including low coverage [12].

Stage distribution of cervical cancer depends on health-seeking behaviour of the population and access to high quality screening and diagnostic services. Proportion of patients registered at stage I or II in the present study (59.5%) was comparable to that observed among the cervical cancer patients registered in Surveillance, Epidemiology and End Results (SEER) database of the USA (59.5%) between 2010 and 2015 [13]. The proportion of late-stage cancer (stage III/IV) was significantly lower in our study than what is generally reported in most LMICs [14]. However, hospital-based studies like ours may overestimate the proportion of early-stage cancer and not reflect the situation among general population. Unexpectedly, parity was found to be a protective factor in diagnosis of late stage cervical cancer in our study, whereas previous studies have shown that parity is associated with late presentation of cervical cancer in the sub Saharan Africa setting [15]. Moroccan women generally have more contact with the primary healthcare system, as they are traditionally the primary caregivers for their children [16]. This could probably explain our results, assuming that women tend to seek care along with their children.

Implementation of screening programme is expected to achieve downstaging of cancer with time, which was not observed in Morocco. As mentioned earlier, the screening programme in the country was not of desirable quality. In fact, higher proportion of advanced stage disease was seen among patients registered in 2013–17 compared to those registered in 2008–12. Most likely explanations are better diagnostic work up of the patients with more extensive use of CT and PET scan and a change in referral practice. More patients with advanced stage disease were referred from periphery to these centers for brachytherapy as the awareness among gynecologists and oncologists grew over time.

Efficiency of a health system to provide quality oncology care is assessed by measuring the delays in care pathway (access delay, diagnostic delay, and treatment delay). WHO recommends that the interval between symptom onset and treatment initiation for cancer patients should not exceed 3 months [17]. We could measure all the delays except diagnostic delay in our study. The access and treatment delays for cervical cancer patients in Morocco (median 5 months and 2.3 months respectively) are still too long compared to the WHO specified standard [12]. The awareness campaigns and improvement of cancer diagnostic services associated with the cervical cancer screening programme may have achieved marginal reduction of access delay over time.

There are several national and international guidelines for stage-appropriate management of cervical cancer. A recent systematic review of post-treatment follow-up studies observed that the low adherence to guidelines in the treatment of cervical cancers was a global problem; the proportion adhering to guidelines among published studies ranged from 42 to 54% only [18]. A retrospective analysis of impact of compliance to clinical practice guidelines reported a significantly higher 5-year survival in cervical cancer patients treated according to the guidelines compared to those that were not, and the benefit of adherence to guidelines being more in stage I/II cancers [19]. The results of our study in Morocco support the observations of earlier studies.

Stage I and II cervical cancers with small lesions and negative nodal metastases are treated with either radical hysterectomy (with bilateral pelvic lymphadenectomy) or radiotherapy. Randomized controlled trials have demonstrated that patients with stage IB and IIA cervical cancer have same overall and disease-free survivals irrespective of whether they are treated by radical surgery or RT [20]. However, a combination of both the modalities is to be avoided as much as possible, since complication rates are significantly higher with combined treatment [21]. Radical surgery was offered to a significant proportion of patients belonging to stage I (81.3%) and stage II (34.8%) in our study. A very high proportion (85.7%) of these patients also received RT, which could be reduced with better diagnostic workup and more careful case selection for radical surgery.

The National Cancer Institute (NCI) of the USA made a clinical announcement in 1999 recommending the use of chemoradiation for all cervical cancer patients undergoing RT [22]. This announcement was based on the outcomes of three randomized trials published almost simultaneously demonstrating nearly 50% improvement in survival with chemoradiation [23–26]. In Morocco approximately half of the patients receiving RT did not receive concomitant CT. Concurrent chemotherapy with radiation being a routine practice at both CM-VI and INO to treat cervical cancer during entire study period, the oncologists at both the centers felt that the proportion of patients reported to have missed chemotherapy was too high. They ascribed the discrepancy to issues related to documentation in the case records. Nevertheless, there is a major scope of improving the proportion of patients treated with chemoradiation in Morocco, especially because both cisplatin and carboplatin (the drugs of choice for chemoradiation) are included in the updated list of essential medicines in the country, thus facilitating their procurement by the public hospitals.

Total duration of radiotherapy is an important quality indicator. Entire course of radiation (EBRT plus brachytherapy) is recommended to be completed within 8 weeks to optimize treatment benefits [27]. Each extra day of overall treatment time can reduce the cause specific survival by 0.5% to 1% [28, 29]. A high proportion of cervical cancer patients in Morocco completed their RT in less than 10 weeks. However, the proportion of patients requiring more than 10 weeks to complete radiation at CM-VI and INO was significantly higher in 2013–17 (38.5%) compared to 2008–12 (14.9%), which is a matter of concern. A discussion with the oncologists at both the centers revealed that the treatment time increased with increased caseloads for RT.

Brachytherapy is a key component of RT. HDR brachytherapy capable of treating 10–12 patients per day is a very useful facility for LMICs with high cervical cancer burden. Studies have shown that women with locally advanced cervical cancer treated with brachytherapy along with EBRT have lower complication rates and better survival compared to women treated with EBRT alone [30, 31]. We observed that brachytherapy was underutilized at the oncology centers in Morocco and not treating patients with brachytherapy significantly compromised survival of these patients. There were periodic issues with maintenance of the brachytherapy machines that led to interruptions in treatment, especially at CM-VI. Patients were referred to other centers for brachytherapy when such problems occurred. Many of these patients did not receive brachytherapy as the facility was available at limited number of public hospitals outside these two oncology centers.

Our study clearly demonstrates the value of having comprehensive oncology care facilities within a single institution. Many of the patients registered at CM-VI had their surgery done at University Teaching Hospital, a non-oncology tertiary care facility. The patients often had to wait for a long time to get their diagnostic work up done at the teaching hospital before initiating radiation and chemotherapy. There was no prioritization of the cancer patients. This was not the case at INO, which was equipped with all facilities. The difference in care at the two oncology centers is reflected in the difference in DFS. Five year DFS was much higher at INO compared to CM-VI, even though the proportion of advanced stage cancers was higher in the former.

Our study has several limitations. The data collected from the two oncology centers based in two major cities cannot be extrapolated to the national context and certainly do not reflect the general standard of care of cervical cancer patients across Morocco. We selected the two Institutions because the Ministry of Health made special investments to improve breast and cervical cancer treatment in these facilities. Our study highlights what best has been achieved in Morocco and despite the efforts the huge scope of improvement that is still there. Any retrospective record-based study like ours has the inherent limitation of incomplete data collection. Even though quality of recordkeeping was generally high at the oncology centers, some key information like completion of chemotherapy or brachytherapy outside the centers were missing. Information on deaths was rarely available, a limitation that precluded estimation of overall survival.

## Conclusion

To conclude, despite many progressive steps taken by the Ministry of Health to improve cervical cancer early detection and management, there are several deficiencies that need to be addressed. The 5-year disease-free survival of 57.5% observed in our study for cervical cancer was higher than that usually reported from other LMICs; still there is room for significant improvement through reduction in delays in care and better adherence to internationally recommended treatment guidelines [14]. All early-stage cervical cancer patients need to be appropriately investigated before being subjected to radical surgery. A new national guideline for investigation and management of cervical cancer patients taking into consideration the FIGO 2018 staging recommendations has been prepared by the Ministry of Health of Morocco [32]. The new guideline needs to be widely disseminated. All health professionals involved in cervical cancer management require orientation training to ensure high adherence to the new guidelines. Increasing the hours of radiation therapy services can absorb the extra caseload. Maintenance of high-technology radiotherapy machines is always a challenge in the LMICs. Having a trained and better equipped maintenance team can reduce the machine downtime. The patients need to be appropriately navigated while being referred to other centers due to faults in the radiotherapy machines. These steps will permit Morocco consolidate the gains that have already been made from the progressive actions and allow the country remain aligned to cervical cancer elimination strategy of WHO.

## Supplementary Information

**Additional file 1: Table 1. **Independent determinants of presentation in advanced (III-IV) stage atdiagnosis.

## Data Availability

The data that support the findings of this study are available on request from the corresponding author. The data are not publicly available due to privacy or ethical restrictions.

## Abbreviations

CM-VI: Centre Mohammed VI pour le traitement des cancers
DSF: Disease-free survival
EBRT: External beam radiotherapy
FIGO: International Federation of Gynaecologists and Obstetricians
HDR: High dose rate
IARC: International Agency for Research on Cancer
INO: Institut National d’Oncologie Sidi Mohamed Ben Abdellah
JAGS: Just Another Gibbs Sampler
LINAC: Linear accelerators
LMICs: Low- and middle-income countries
MENA: Middle East North Africa
NCI: National Cancer Institute
Ors: Odds ratios
POC: Pattern-of-care
RT: Radiation therapy
SEER: Surveillance, Epidemiology and End Results
WHO: World Health Organization
