# Supplementary material for: Evolution of patterns of care for women with cervical cancer in Morocco over a decade
Source: BMC Cancer. 2022 May 2;22:479. doi: 10.1186/s12885-022-09358-x (PMC9059352; doi:10.1186/s12885-022-09358-x)
Supplement: Supplementary file 1 — Additional file 1: Table 1. Independent determinants of presentation in advanced (III-IV) stage atdiagnosis. [file 12885_2022_9358_MOESM1_ESM.docx]

**Supplementary table 1: Independent determinants of presentation in advanced (III-IV) stage at diagnosis**

| **Characteristics** | **Patients** | **Patients with** | | **Crude odds ratio** | | |  | **Adjusted odds ratio** | | |
| --- | --- | --- | --- | --- | --- | --- | --- | --- | --- | --- |
|  | **assessed** | **advanced** | | **(95% CI)^a^** | | |  | **(95% CI)^a,b^** | | |
|  |  | **(III-IV) stage** | |  | | |  |  | | |
|  | **n** | **n (%)** | |  | | |  |  | | |
| Patients with symptoms | 787 | 319 | (40.5) |  |  |  |  |  |  |  |
| Centre |  |  |  |  |  |  |  |  |  |  |
| CM-VI, Casablanca | 318 | 113 | (35.5) | 1.00 |  |  |  | 1.00 |  |  |
| INO, Rabat | 469 | 206 | (43.9) |  |  |  |  |  |  |  |
| Period of diagnosis |  |  |  |  |  |  |  |  |  |  |
| 2008-2012 | 476 | 178 | (37.4) | 1.00 |  |  |  | 1.00 |  |  |
| 2013-2017 | 311 | 141 | (45.3) | 1.38 | (1.03 - | 1.83) |  | 1.39 | (1.00 - | 1.83) |
| Age at diagnosis (years) |  |  |  |  |  |  |  |  |  |  |
| <50 | 244 | 87 | (35.7) | 1.00 |  |  |  | 1.00 |  |  |
| 50-59 | 266 | 113 | (42.5) | 1.34 | (0.91 - | 1.88) |  | 1.35 | (0.82 - | 1.97) |
| 60+ | 277 | 119 | (43.0) | 1.38 | (0.91 - | 1.92) |  | 1.46 | (0.84 - | 2.28) |
| Residence |  |  |  |  |  |  |  |  |  |  |
| Urban | 528 | 221 | (41.9) | 1.00 |  |  |  | 1.00 |  |  |
| Semi-urban | 82 | 28 | (34.1) | 0.71 | (0.41 - | 1.11) |  | 0.77 | (0.44 - | 1.23) |
| Rural | 177 | 70 | (39.5) | 0.93 | (0.63 - | 1.28) |  | 1.03 | (0.69 - | 1.44) |
| Parity |  |  |  |  |  |  |  |  |  |  |
| 0-2 | 174 | 88 | (50.6) | 1.00 |  |  |  | 1.00 |  |  |
| 3-4 | 202 | 79 | (39.1) | 0.63 | (0.39 - | 0.91) |  | 0.62 | (0.39 - | 0.90) |
| 5+ | 359 | 134 | (37.3) | 0.59 | (0.39 - | 0.82) |  | 0.49 | (0.32 - | 0.71) |
| Menopause status |  |  |  |  |  |  |  |  |  |  |
| No | 248 | 87 | (35.1) | 1.00 |  |  |  | 1.00 |  |  |
| Yes | 474 | 207 | (43.7) | 1.44 | (0.99 - | 1.91) |  | 1.27 | (0.79 - | 1.86) |
| Access delay |  |  |  | 1.02 | (1.00 - | 1.03) |  | 1.02 | (1.00 - | 1.03) |
| CI: confidence interval; CM-VI: Centre Mohammed VI pour le traitement des cancers ; INO: Institut National d'Oncologie Sidi Mohamed Ben Abdellah ; ^a^ adjusted for clustering within the centre; ^b^ all listed variables included in the adjusted regression model | | | | | | | | | | |
